# Socioeconomic impact of multiresistant nosocomial infections – preliminary results of a qualitative study

**DOI:** 10.1186/2047-2994-4-S1-P103

**Published:** 2015-06-16

**Authors:** Y Mo, PA Tambyah

**Affiliations:** 1 Infectious Disease, National University Hospital Singapore, Singapore, Singapore

## Introduction

Multiresistant nosocomial infections have been extensively studied. However, there are few data on these infections from the patients' perspectives.

## Objectives

The study aims to explore the patients' in-depth experiences about multiresistant nosocomial infections, and the socioeconomic impacts.

## Methods

The study was conducted in the National University Hospital, Singapore, a tertiary referral hospital with approximately 1200 beds. The study includes patients who have acquired multiresistant nosocomial infections who agreed to participate in extended interviews.

## Results

Six patients were interviewed between April and May 2010. All the patients suffered deep-seated infections caused by multiresistant organisms including Methicillin-resistant *Staphylococcus aureus* (MRSA), extended-spectrum beta-lactamases (ESBL) producing gram-negative bacteria.

All the patients had poor knowledge in the sources of infections, symptoms, modes of transmission, prevention, and complications. The commonest misconceptions included ideas that nosocomial infections are mostly airborne, infections are expected consequences of most sickness, and taking ‘strong’ antibiotics can prevent infections. These perceptions were common to patients regardless of the duration of illness and education levels.

These patients also displayed somewhat nihilistic attitudes towards their illness. They did not feel that having more involvement in their management would impact their state of health. A common barrier was the fear that their care could be compromised if they questioned the healthcare professionals.

5 out of 6 patients felt that the infections caused a significant financial impact, which included loss of monthly salaries, cancellation of financial commitments, and compromise in future career prospects.

The patients interviewed did not face any social stigma associated with nosocomial multiresistant infections despite the use of contact precautions in hospital.

## Conclusion

Patients continue to have little knowledge and involvement in the management of their health in the nosocomial infections. With the increasing education standards across the globe, more needs to be done in educating the public about multiresistant organisms and infections to achieve a comprehensive approach to their prevention and control.

## Disclosure of interest

None declared.

